# The complete mitochondrial genome of the *Opisthotropis guangxiensis* (Squamata: Colubridae)

**DOI:** 10.1080/23802359.2020.1789515

**Published:** 2020-07-15

**Authors:** Jing Zhang, Dan Wang

**Affiliations:** a West China School of Medicine, West China Hospital, Sichuan University, Chengdu, Sichuan, China; b Chengdu Institute of Biology, Chinese Academy of Sciences, Chengdu, Sichuan, China

**Keywords:** Complete mitochondrial genome, *Opisthotropis guangxiensis*, Colubridae, phylogenetic

## Abstract

In this study, we report the complete mitochondrial genome of the *Opisthotropis guangxiensis*, which is 17,042 bp in size and includes 13 protein-coding genes (PCGs), 22 tRNA genes, 2 rRNA genes, and 2 non-coding regions. The sequence presented would be useful for better understanding the phylogenetic and evolutionary of genus *Opisthotropis.*

The genus *Opisthotropis*, which inhabits flowing streams or waterfalls, is distributed across southern China and Southeast Asia (Pope 1935; Zhao 2006). *Opisthotropis guangxiensis* belongs to the genus *Opisthotropis*, mainly distributed in Guangxi Autonomous Region, China (Zhao and Adler 1993). In this study, a complete mitochondrial genome of *O. guangxiensis* was reported.

The specimens were collected from Guangxi Autonomous Region, China and deposited at herpetological museum, Chengdu Institute of Biology, CAS with the number CIB116957. We used the liver tissue to extract the total genomic DNA using EasyPure@ Genomic DNA Kit according to the manufacturer’s instructions (TransGen Biotech Co., Beijing, China). The complete mitochondrial genome of *O. guangxiensis* was generated from the next-generation sequencing data. The Mitochondrial genome annotation web server (MITOS) was used for annotation (Bernt et al. 2013). The tRNA genes were scanned by tRNAScan-SE2.0 online website (http://lowelab.ucsc.edu/tRNAscan-SE/) (Lowe and Chan 2016).

The complete and circular mitochondrial genome is 17,042 bp in size, with base compositions of 34.9% A, 25.8% T, 12.5% G, and 26.7% C. The mitochondrial genome contained 13 protein-coding genes (PCGs) (11,325 bp, ND1-6, ND4L, CO1-3, Cytb, ATP6, and ATP8), two rRNA genes (12s and 16s rRNA), 22 tRNA genes, and 2 non-coding regions (CR and D-loop). All the PCGs use ATN as the start codon except COX1 (GTG) and ATP8 (GTG); ATP8, ND4L, ND4, and ND5 use typical stop codon TAA. Among these genes, 28 of 37 genes are encoded on the H-strand, containing 12 PCGs, 2 rRNA, 14 tRNA; and other nine genes are located on the L-strand, containing ND6 and 8 tRNA. For the 13 PCGs, the longest gene is ND5 (1782 bp), while the shortest gene is ATP8 (162 bp). The 22 tRNA genes varied in size from 57 to 73 bp. The mitogenome of *O. guangxiensis* has been deposited in GenBank under accession number MT571495.

A maximum-likelihood (ML) tree (Figure 1) of *O. guangxiensis* and other 11 snakes was reconstructed based on 12 H-strand PCGs using IQ-TREE (Lam-Tung et al. 2015). The alignment was performed by MEGA7 (Kumar et al. 2016) with default settings. *Bungarus fasciatus* was selected as outgroup. The results showed that the *O. guangxiensis* and *Opisthotropis latouchii* formed sister groups, and they were closely related with *Rhabdophis tigrinus.*

**Figure 1.**
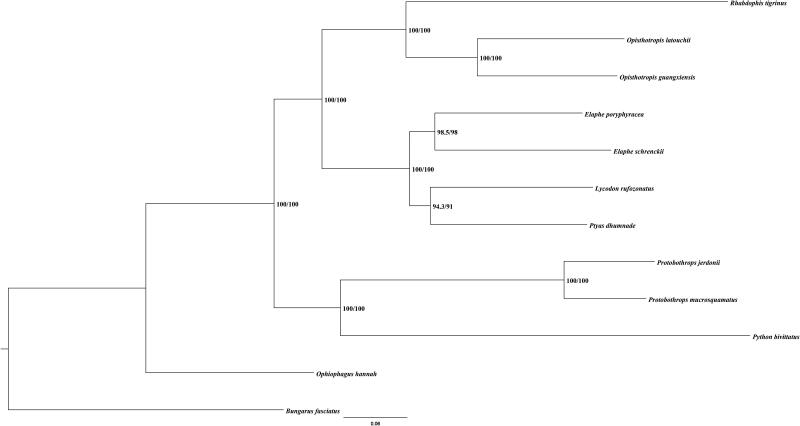
Phylogenetic tree of the relationships among snakes based on ML method. Numbers in parentheses are SH-aLRT support (%)/ultrafast bootstrap support (%). The species selected and corresponding GenBank accession number are shown as follows: *Bungarus fasciatus* (NC_011393), *Elaphe poryphyracea* (NC_012770), *Elaphe schrenckii* (NC_027605), *Lycodon rufozonatus* (NC_024559), *Ophiophagus hannah* (NC_011394), *Opisthotropis latouchii* (NC_046823), *Protobothrops jerdonii* (NC_021402), *Protobothrops mucrosquamatus* (NC_021412), *Ptyas dhumnades* (NC_028049), *Python bivittatus* (NC_021479), and *Rhabdophis tigrinus* (NC_030210).

## Data Availability

The data that support the findings of this study are openly available in “NCBI” at https://www.ncbi.nlm.nih.gov/, reference number MT571495.
